# Repeated *Aspergillus* isolation in respiratory samples from non-immunocompromised patients not selected based on clinical diagnoses: colonisation or infection?

**DOI:** 10.1186/1471-2334-12-295

**Published:** 2012-11-12

**Authors:** Jose Barberan, Bernardino Alcazar, Eduardo Malmierca, Francisco Garcia de la Llana, Jordi Dorca, Daniel del Castillo, Victoria Villena, Melissa Hernandez-Febles, Francisco-Javier Garcia-Perez, Juan-Jose Granizo, Maria-Jose Gimenez, Lorenzo Aguilar

**Affiliations:** 1 Infectious Diseases Department, Hospital Central de la Defensa Gómez Ulla, Gta. del Ejército s/n, 28047, Madrid, Spain; 2 Pneumology Department, Complejo Hospitalario de Jaen, Jaen, Spain; 3 Internal Medicine Department, Hospital Infanta Sofia, San Sebastián de los Reyes, Madrid, Spain; 4 Infectious Diseases Department, Complejo Hospitalario Universitario de Badajoz, Badajoz, Spain; 5 Pneumology Department, Hospital Universitari de Bellvitge, Institut d’Investigació Biomèdica de Bellvitge (IDIBELL), Barcelona, Spain; 6 Pneumology Department, Hospital de Jerez, Jerez de la Frontera, Cadiz, Spain; 7 Pneumology Department, Hospital Universitario 12 de Octubre, Madrid, Spain; 8 Microbiology Department, Hospital Universitario de Gran Canaria Dr. Negrin, La Palmas de Gran Canaria, Spain; 9 Pneumology Department, Hospital Universitario de la Princesa, Madrid, Spain; 10 Grana Datos, Pozuelo de Alarcón, Madrid, Spain; 11 Microbiology Department, School of Medicine, Universidad Complutense, Madrid, Spain

**Keywords:** Aspergillus, COPD, Clinical management, Aspergillosis

## Abstract

**Background:**

Isolation of *Aspergillus* from lower respiratory samples is associated with colonisation in high percentage of cases, making it of unclear significance. This study explored factors associated with diagnosis (infection vs. colonisation), treatment (administration or not of antifungals) and prognosis (mortality) in non-transplant/non-neutropenic patients showing repeated isolation of *Aspergillus* from lower respiratory samples.

**Methods:**

Records of adult patients (29 Spanish hospitals) presenting ≥2 respiratory cultures yielding *Aspergillus* were retrospectively reviewed and categorised as proven (histopathological confirmation) or probable aspergillosis (new respiratory signs/symptoms with suggestive chest imaging) or colonisation (symptoms not attributable to *Aspergillus* without dyspnoea exacerbation, bronchospasm or new infiltrates). Logistic regression models (step–wise) were performed using Aspergillosis (probable + proven), antifungal treatment and mortality as dependent variables. Significant (p < 0.001) models showing the highest R^2^ were considered.

**Results:**

A total of 245 patients were identified, 139 (56.7%) with Aspergillosis. Aspergillosis was associated (R^2^ = 0.291) with ICU admission (OR = 2.82), congestive heart failure (OR = 2.39) and steroids pre-admission (OR = 2.19) as well as with cavitations in X-ray/CT scan (OR = 10.68), radiological worsening (OR = 5.22) and COPD exacerbations/need for O_2_ interaction (OR = 3.52). Antifungals were administered to 79.1% patients with Aspergillosis (100% proven, 76.8% probable) and 29.2% colonised, with 69.5% patients receiving voriconazole alone or in combination. In colonised patients, administration of antifungals was associated with ICU admission at hospitalisation (OR = 12.38). In Aspergillosis patients its administration was positively associated (R^2^ = 0.312) with bronchospasm (OR = 9.21) and days in ICU (OR = 1.82) and negatively with Gold III + IV (OR = 0.26), stroke (OR = 0.024) and quinolone treatment (OR = 0.29). Mortality was 78.6% in proven, 41.6% in probable and 12.3% in colonised patients, and was positively associated in Aspergillosis patients (R^2^ = 0.290) with radiological worsening (OR = 3.04), APACHE-II (OR = 1.09) and number of antibiotics for treatment (OR = 1.51) and negatively with species other than *A. fumigatus* (OR = 0.14) and aspergillar tracheobronchitis (OR = 0.27).

**Conclusions:**

Administration of antifungals was not always closely linked to the diagnostic categorisation (colonisation vs. Aspergillosis), being negatively associated with severe COPD (GOLD III + IV) and concomitant treatment with quinolones in patients with Aspergillosis, probably due to the similarity of signs/symptoms between this entity and pulmonary bacterial infections.

## Background

Isolation of *Aspergillus* from lower respiratory samples is associated with colonisation in high percentage of cases, being of unclear significance because it may represent a temporary passage, a long-term benign carriage or a sign preceding invasive disease since the incubation period is unknown [1,2]. Invasive disease has been described as disease of immunocompromised patients, and for high risk patients (those with bone marrow or solid organ transplant, neutropenia or haematological cancer [3]) standard definitions of opportunistic infection (proven, probable or possible) to express disease certainty have been internationally agreed [4]. In these type of patients, isolation of *Aspergillus* from lower respiratory tract samples is potentially significant [5], and acquires relevance since early diagnosis seems crucial to improve prognosis of this potentially treatable disease [1,6].

Invasive aspergillosis is increasingly being recognised as an emerging opportunistic infection in non-neutropenic patients, with reports on patients receiving immunossupressive therapy that do not impair neutrophils count, patients with cancer (receiving or not treatment), connective diseases requiring corticosteroids, liver cirrhosis, elderly patients (that comprise a growing segment of hospitalised patients), patients with less immunodeficiency such as patients with chronic obstructive pulmonary disease (COPD), specially those under steroid therapy, and ICU patients without apparent predisposing immunodeficiency [7-12].

Sensitivity of culture isolation for invasive infection varies from 5% to 75%, depending on the population [10]. Since culture has also poor specificity, it has been suggested the need for repeated isolation of the same *Aspergillus* species as part of the diagnosis of invasive aspergillosis [13], in a field where the use of diagnostic tools widely differs between different hospitals.

The aim of this study was to explore implications for the patient’s management of the repeated isolation of *Aspergillus* from lower respiratory samples in non-transplant, non-neutropenic patients not selected based on clinical diagnoses, by means of identification of factors associated with diagnosis (infection vs. colonisation), with treatment (administration of antifungals) and with prognosis (mortality).

## Patients and methods

Clinical records of adult patients presenting at least two cultures of evaluable lower respiratory tract samples yielding *Aspergillus* in 29 Spanish hospitals were retrospectively reviewed to include up to 10 patients in each centre (the 10 most recent consecutive evaluable patients with complete clinical records in the period 2001–2010). Eligible clinical records were identified through microbiology records. Transplant recipients and patients presenting neutropenia (<1000 neutrophils/mm^3^), diagnosis of aspergilloma or allergic bronchopulmonary aspergillosis were excluded. The study protocol was approved by the Ethics Committee of Hospital Central de la Defensa Gomez Ulla, Madrid, Spain.

Demographic data, underlying illnesses, clinical and radiological data, laboratory data, previous corticosteroids intake (within 3 months prior to admission), previous antibiotic and antifungal treatments (within 30 days prior to admission), antifungal treatment during hospitalization and outcome were recorded. The age-unadjusted Charlson comorbidity index [14] (age was considered in separate), the modified McCabe score (Sabadell score) [15], the functional classification according to the New York Heart Association (NYHA) [16] and the Acute Physiologic and Chronic Health Evaluation (APACHE) II score were calculated with recorded data, as well as the Global Initiative for Chronic Obstructive Lung Disease (GOLD) classification for COPD patients [17]. A diagnostic category was assigned to each patient using criteria adapted from those defined by Bulpa et al. for COPD patients [1], not considering the “possible” category since in the present study all patients required at least two positive *Aspergillus* cultures as inclusion criteria. Patients were classified as with proven aspergillosis (histopathological confirmation), probable aspergillosis (presence of new respiratory signs/symptoms with suggestive chest imaging) or colonisation (symptoms not attributable to *Aspergillus* without dyspnoea exacerbation, bronchospasm or new pulmonary infiltrates). For data analysis a category named “Aspergillosis” was considered by pooling patients with probable aspergillosis and proven aspergillosis. Diagnoses of tracheobronchitis were always based on bronchoscopy reports included in clinical records. Simple tracheobronchitis was considered when the bronchoscopy report described mucosal inflammation and mucus secretions and invasive tracheobronchitis when ulceration and pseudomembrane formation was observed. Chronic pulmonary aspergillosis was considered when long-term fibrotic lesions with or without necrosis or cavitations had been recorded in the patient’s clinical records.

Comparisons between proportions were performed by the χ^2^ test and the Fisher’s exact test, when necessary. For quantitative variables, since data did not showed normality in the Kolmogorov – Smirnoff test, the Kruskal-Wallis and Mann–Whitney tests, when necessary, were used. Bivariate analyses were performed comparing all variables between colonised vs. patients with Aspergillosis, between antifungal treated vs. non-treated patients and between patients who died vs. those that did not. Different logistic regression models (step–wise procedure) were performed using as dependent variables i) Aspergillosis, ii) antifungal treatment and iii) mortality, and as independent variables those showing differences (p ≤ 0.1) in bivariate analyses. Interactions and linear dependence between independent variables were previously controlled. Statistical analyses were performed using SPSS v 14 programme (SPSS Inc, Chicago, IL). The models showing the highest R^2^ were considered.

## Results

A total of 245 clinical records were included in the study, corresponding to patients attended in the study centres from October 2002 to July 2010 (19 centres included 10 patients each and 10 centres included the remaining 55 patients). Of the 245 patients considered, 106 were categorised as colonised and 139 as patients with Aspergillosis (125 as probable and 14 as proven aspergillosis). Six patients (2.4%) had cystic fibrosis (5 patients classified as colonized and 1 as with probable aspergillosis). *Aspergillus fumigatus* was the most frequent species isolated, accounting for 66.9% of total isolates, followed by *Aspergillus niger* in 6.1% patients and *Aspergillus flavus* in 3.7% patients. In a total of 23.3% patients cultures had been informed as *Aspergillus* spp. or other species. *A. fumigatus* was isolated in all cases categorised as proven aspergillosis. The serum galactomannan level had been determined in 51 (20.8%) patients. The result was positive (≥1 ng/ml) in 33.3% (14 out of 42) patients with Aspergillosis while it was negative in all patients categorised as colonised in which the test was performed (n = 9). Histopathological examination was performed in 19 patients, the 14 patients classified as with proven aspergillosis and another 5 patients where biopsies were performed and were negative (patients classified as with probable aspergillosis). In the 14 patients with aspergillosis, histopathological confirmation was obtained by fine needle aspiration biopsy (2 patients), pulmonary biopsy (8 patients), renal biopsy (1 patient) and necropsy (3 patients).

### Descriptive analysis

Table 1 shows epidemiological data and comorbidities present in the study patients distributed by diagnostic category. Mean age of patients was 68.7 ± 15.2 years (with 42.9% patients ≥75 years of age and 22.0% ≥80 years), without differences between categories. The percentage of males was significantly higher among patients with Aspergillosis (74.8% vs. 63.2% in colonised; p = 0.05). Significantly higher percentage of patients with severe COPD (GOLD III + IV) (p = 0.037), congestive heart failure (p = 0.031) and malignancies (p = 0.011) were found among patients with Aspergillosis, without significant differences in the Charlson index between categories. The mean APACHE II score was significantly higher in patients with Aspergillosis (14.33 vs. 11.63 in colonised; p = 0.004), reaching a value of 16.14 in patients with proven aspergillosis. Overall prognosis (McCabe score) was non fatal in 60.0% patients, long-term fatal (>6 months) in 31.0% patients and short-term fatal (<6 months) in 8.9% patients, without differences between categories.

**Table 1:** Epidemiological data and comorbidities of patients included in the study distributed by diagnostic category; [n (%)] except where indicated

	**Total**	**Colonised**	**Aspergillosis**		
			**Probable**	**Proven**	**Probable + Proven**
**No. Patients**	245	106	125	14	139
**Age (mean** ± **SD)**	68.7 ± 15.2	69.1 ± 16.1	68.9 ± 14.6	64.1 ± 14.5	68.4 ± 14.6
**Males**	171 (69.8)	67 (63.2)	94 (75.2)	10 (71.4)	104 (74.8)^a^
**Comorbidities**^**b**^					
**COPD**	173 (70.6)	73 (68.9)	91 (72.8)	9 (64.3)	100 (71.9)
**Gold III + IV**^c^	111 (67.3)	40 (57.2)	66 (76.7)^a^	5 (55.6)	71 (74.7)^a^
**Diabetes Mellitus II**	43 (17.6)	19 (17.9)	23 (18.4)	1 (7.1)	24 (17.3)
**Congestive heart failure**	45 (18.4)	13 (12.3)	27 (21.6)	5 (35.7)^a^	32 (23.0)^a^
**Malignancies**	38 (15.5)	10 (9.4)	27 (21.6)^a^	1 (7.1)	28 (20.1)^a^
**Renal impairment**	24 (9.8)	10 (9.4)	12 (9.6)	2 (14.3)	14 (10.1)
**Charlson (mean** ± **SD)**	2.61 ± 2.16	2.42 ± 2.03	2.81 ± 2.29	2.21 ± 1.76	2.75 ± 2.75
**APACHE (mean** ± **SD)**	13.16 ± 6.86	11.63 ± 6.12	14.13 ± 6.67^a^	16.14 ± 10.90	14.33 ± 7.18^a^
**ICU admission at any time during hospital stay**	58 (23.7)	15 (14.2)	36 (28.8)^a^	7 (50.0)^a^	43 (30.9)^a^

^a^p ≤ 0.050 vs. colonised; ^b^present in ≈ > 10% total patients; ^c^among patients with available data (165 patients in total: 70 colonized, 86 probable and 9 proven).

Only 18 patients (7.3%) were ambulatory, 72.2% categorised as colonised and 27.8% as with probable aspergillosis. A total of 48.2% patients had been initially admitted in Pneumology, 23.3% in Internal Medicine, 11.4% in the Intensive Care Unit (ICU) and 9.8% in other wards, without differences between diagnostic categories. Admission in the ICU as initial ward was more frequent among proven cases (28.6%) than among probable (11.2%) or colonised cases (9.4%), although differences did not reach statistical significance (p = 0.085 vs. probable and p = 0.058 vs. colonised). ICU admission at any time during hospitalisation (Table 1) was significantly higher among patients with Aspergillosis (30.9%) than among those colonised (14.2%) (p = 0.001), with 50.0% admission rate among those with proven aspergillosis (p = 0.004 vs. colonised) and 28.8% among those with probable aspergillosis (p = 0.007 vs. colonised).

Table 2 shows imaging data of patients distributed by diagnostic categories. Significantly (p < 0.001) higher percentage of patients showing infiltrates and cavitations was found (both in X-ray and CT scan) in the group of patients with Aspergillosis than in colonised patients. Worsening of radiological findings in successive X-ray was also significantly more frequent among patients with Aspergillosis (36.7% vs. 6.7% colonised, p < 0.001). CT scan was more frequently requested in patients further categorised as having Aspergillosis than in those categorised as colonised (64.7% vs. 46.2%, p = 0.004), being up to 78.6% among patients with proven aspergillosis. The halo and the air crescent signs were only found in 4 and 3 cases, respectively, of Aspergillosis.

**Table 2:** Imaging data corresponding to patients included in the study distributed by diagnostic category; [n (%)]

	**Total**	**Colonised**	**Aspergillosis**					
			**Probable**	**Proven**	**Probable + Proven**			
**No. patients with chest Rx**	244	105	125	14	139
**Infiltrates**	134 (54.9)	46 (43.8)	76 (60.8)^a^	12 (85.7)^a^	88 (63.3)^a^
**Nodules**	37 (15.2)	13 (12.4)	24 (19.2)	0 (0.0)	24 (17.3)
**Cavitations**	21 (8.6)	0 (0.0)	19 (15.2)^a^	2 (14.3)^a^	21 (15.1)^a^
**Pleural effusion**	25 (10.2)	7 (6.7)	16 (12.8)	2 (14.3)	18 (12.9)
**Worsening radiological findings**	58 (23.8)	7 (6.7)	43 (34.4)^a^	8 (57.1)^a^	51 (36.7)^a^
**No. patients with CT Scan**	139 (56.7)	49 (46.2)	79 (63.2)^a^	11 (78.6)^a^	90 (64.7)^a^
**Infiltrates**	78 (56.1)	19 (39.6)	48 (60.0)^a^	11 (100)^a^	59 (64.8)^a^
**Nodules**	56 (40.3)	14 (28.6)	39 (49.4)^a^	3 (27.3)	42 (46.7)^a^
**Cavitations**	33 (23.7)	5 (10.2)	23 (29.1)^a^	5 (45.5)^a^	28 (31.1)^a^
**Halo sign**	4 (2.9)	0 (0.0)	3 (3.8)	1 (9.1)	4 (4.4)
**Air crescent sign**	3 (2.2)	0 (0.0)	3 (3.8)	0 (0.0)	3 (3.3)

^a^p ≤ 0.05 vs. colonised.

Table 3 shows intake of steroids, antibiotics and antifungals dividing patients by diagnostic categories. Prior to admission, the percentage of patients with Aspergillosis that had received corticosteroids was significantly higher than those colonised (51.8% vs. 27.4%, p < 0.001) due to the subgroup of patients receiving accumulated doses >100 mg (81.9% vs. 58.6%, p = 0.033). During hospitalisation, 75.5% patients received steroids without differences between diagnostic categories. A total of 35.9% patients received antibiotics prior to admission and 82.0% received them during hospitalisation, without differences between diagnostic categories in both cases. Antifungal treatment was initiated after culture request in 79.1% patients further categorised as having Aspergillosis (100% proven cases and 76.8% probable cases) and in 29.2% colonised patients (p < 0.001). Voriconazole was the most frequent antifungal used, with up to 69.5% patients receiving this compound alone or in combination with other antifungals. In patients with Aspergillosis, antifungal treatment was voriconazole as monotherapy in 51.8% cases, voriconazole plus other antifungals in 22.7% cases and other antifungals (amphotericin, caspofungin, fluconazole and itraconazole) alone or in combination in 25.5% cases.

**Table 3:** Steroids, antibiotic and antifungals intake [n (%)] by diagnostic category

	**Total**	**Colonised**	**Aspergillosis**		
			**Probable**	**Proven**	**Probable + Proven**
**No. patients**	245	106	125	14	139
**Steroids intake before admission**	101 (41.2)	29 (27.4)	64 (51.2)^a^	8 (57.1)^a^	72 (51.8)^a^
**≥20 mg/day**	59 (58.4)	15 (51.7)	40 (62.5)	4 (50.0)	44 (61.1)
**Accumulated dose >100 mg**	76 (75.2)	17 (58.6)	52 (81.3)^a^	7 (87.5)	59 (81.9)^a^
**Accumulated dose >700 mg**	20 (19.8)	6 (20.7)	11 (17.2)	3 (37.5)	14 (19.4)
**Antibiotic treatment before admission**	88 (35.9)	34 (32.1)	47 (37.6)	7 (50.0)	54 (38.8)
**Antifungal treatment after culture request**	141 (57.6)	31 (29.2)	96 (76.8)^a^	14 (100)^a^	110 (79.1)^a^
**Voriconazole as monotherapy**	68 (48.2)	11 (35.5)	55 (57.3)	2 (14.3)	57 (51.8)
**Voriconazole + other antifungals**	30 (21.3)	5 (16.1)	18 (18.8)	7 (50.0)	25 (22.7)
**Total patients receiving voriconazole**	98 (69.5)	16 (51.6)	73 (76.1)	9 (64.3)	82 (74.5)
**Other antifungals**	43 (30.5)	15 (48.4)	23 (24.0)	5 (35.7)	28 (25.5)

^a^p ≤ 0.05 vs. colonised.

Table 4 shows clinical presentations and outcome by diagnostic category. A total of 54.6% patients with Aspergillosis presented invasive pulmonary aspergillosis, 18.4% simple tracheobronchitis, 16.3% chronic pulmonary aspergillosis and 8.5% invasive tracheobronchitis. All 14 patients with proven aspergillosis presented pulmonary infection: 71.4% invasive aspergillosis and 28.6% chronic aspergillosis. Significantly higher mortality was found in proven (78.6%) than in probable aspergillosis (41.6%) (p = 0.018) or colonisation (12.3%) (p < 0.001), as well as in probable aspergillosis vs. colonisation (p < 0.001). *Aspergillus* was involved as cause of death (with or without others) in 78.5% patients with proven aspergillosis compared with 32.0% in those with probable aspergillosis (p = 0.002) and 0% in those colonised (p < 0.001).

**Table 4:** Clinical presentations and outcome by diagnostic category

	**Total**	**Colonised**	**Aspergillosis**		
			**Probable**	**Proven**	**Probable + Proven**
**No. patients**	245	106	125	14	139
**Invasive pulmonary aspergillosis**	77 (31.4)		68 (54.4)	10 (71.4)	78 (56.1)
**Chronic pulmonary aspergillosis**	23 (9.4)		19 (15.2)	4 (28.6)	23 (16.5)
**Simple tracheobronchitis**	26 (10.6)		26 (20.8)	0 (0.0)	26 (18.7)
**Invasive tracheobronchitis**	12 (4.9)		12 (9.4)	0 (0.0)	12 (8.6)
**Mortality**	76 (31.0)	13 (12.3)	52 (41.6)^a^	11 (78.6)^a,b^	63 (45.3)^a^
**a) Death attributable to*Aspergillus***	7 (2.9)	0 (0.0)	4 (3.2)	3 (21.4)^a, b^	7 (5.0)
**b) Death attributable to*Aspergillus*and to other causes**	44 (18.0)	0 (0.0)	36 (28.8)^a^	8 (57.1)^a^	44 (31.7)^a^
**a) + b)**	51 (20.9)	0 (0.0)	40 (32.0)^a^	11 (78.5)^a, b^	51 (36.7)
**Death not attributable to*Aspergillus***	25 (10.2)	13 (12.3)	12 (9.6)	0 (0.0)	12 (8.6)

Results are expressed as “n (%)”

^a^p < 0.001 vs. colonised; ^b^p < 0.02 vs. probable.

### Factors defining infection vs. colonisation

In the multivariate analysis exploring variables associated with diagnostic categories (colonisation vs. Aspergillosis), which included all variables showing significance (p < 0.1) in the bivariate analysis previously performed, the logistic regression was statistically significant (p < 0.001, R^2^ Cox = 0.291). Aspergillosis was associated with ICU admission at any time (p = 0.012; OR = 2.82, 95% CI = 1.26-6.37), congestive heart failure (p = 0.044; OR = 2.39, 95% CI = 1.03-5.55) and the intake of steroids prior to admission (p = 0.021; OR = 2.19, 95%CI = 1.13-4.27) in addition to variables suggesting Aspergillosis as the presence of cavitations in X-ray and/or CT scan (p < 0.001; OR = 10.68, 95% CI = 3.61-31.64), the worsening of radiological findings (p = 0.001; OR = 5.22, 95% CI = 2.04-13.33) and the interaction between COPD exacerbations and need for O_2_ (p < 0.001; OR = 3.52, 95% CI = 1.75-7.11).

### Variables associated with administration of antifungals

Table 5 shows variables with statistical significance (p < 0.1) in the bivariate analysis performed comparing patients treated with antifungals vs. those not treated, considering the total population, colonised patients and patients with Aspergillosis. The multivariate analysis performed including the variables shown in the Table arose the following results:

a) Total population The multivariate analysis was statistically significant (p < 0.001, R^2^ Cox = 0.221) and antifungal treatment was associated with stay in the ICU (p < 0.001; OR = 4.78, 95% CI = 2.20-10.36) and the presence of two or more radiological findings (infiltrates and/or nodules and/or cavitations) in X-ray and/or CT scan (p < 0.001; OR = 2.43, 95% CI = 1.67-3.53) and negatively associated with severe liver disease (p = 0.015; OR = 0.15, 95% CI = 0.03-0.69).

b) Patients categorised as colonised In the multivariate analysis the logistic regression was significant (p = 0.001) but showed a weak association (R^2^ Cox = 0.118).Antifungal treatment was only associated with ICU admission at hospitalization (p = 0.002; OR = 12.38, 95% CI = 2.44-62.92).

c) Patients categorised as with Aspergillosis The multivariate analysis was statistically significant (p < 0.001, R^2^ Cox = 0.312) and antifungal treatment was positively associated with days of stay in the ICU (p = 0.021; OR = 1.82, 95% CI = 1.09-3.05) and presence of bronchospasm (p = 0.001; OR = 9.21, 95% CI = 2.63-32.21) and negatively associated with GOLD III + IV (p = 0.024; OR = 0.26, 95% CI = 0.08-0.84), stroke (p = 0.009; OR = 0.024, 95% CI = 0.01-0.40) and concomitant treatment with quinolones (p = 0.034; OR = 0.29, 95% CI = 0.09-0.91).

**Table 5:** Significant variables in the bivariate analysis of patients receiving vs. not receiving antifungals, [n (%)] except where indicated

	**Total patients (n = 245)**			**Colonised (n = 106)**			**Aspergillosis (n = 139)**		
	**Treated (n = 104)**	**Non-treated (n = 141)**	**p**	**Treated (n = 31)**	**Non-treated (n = 75)**	**p**	**Treated (n = 110)**	**Non-treated (n = 29)**	**p**
**Age (X** ± **SD)**	66.7 ± 15.9	71.4 ± 13.8	**0.016**	66.0 ± 16.7	70.4 ± 15.7	0.086	66.9 ± 15.8	73.9 ± 6.6	**0.001**
**Days to isolation (X** ± **SD)**	12.3 ± 12.2	7.6 ± 7.6	**0.001**	8.9 ± 6.6	7.0 ± 6.5	0.096	13.2 ± 13.1	9.1 ± 9.6	**0.046**
**COPD**	90 (63.8)	83 (79.8)	**0.007**	15 (48.4)	58 (77.3)	**0.005**	75 (68.2)	25 (86.2)	0.064
**GOLD III + IV**	57 (40.4)	54 (51.9)	0.074	8 (53.4)	32 (58.2)	0.568	49 (65.3)	22 (88.0)	**0.041**
**COPD exacerbation**	22 (15.6)	3 (2.9)	0.625	6 (19.4)	29 (38.7)	0.070	56 (50.9)	20 (69.0)	0.082
**Severe liver disease**	3 (2.1)	8 (7.7)	0.058	1 (3.2)	6 (8.0)	0.671	2 (1.8)	2 (6.9)	0.192
**Stroke**	4 (2.8)	6 (5.8)	0.332	1 (3.2)	2 (2.7)	1.000	3 (2.7)	4 (13.8)	**0.035**
**APACHE II (X** ± **SD)**	13.8 ± 7.6	12.3 ± 5.6	0.076	12.6 ± 7.5	11.2 ± 5.4	0.291	14.1 ± 7.6	15.1 ± 5.1	0.439
**ICU admission**	47 (33.3)	11 (10.6)	**0.001**	10 (32.3)	5 (6.7)	**0.001**	37 (33.6)	6 (20.7)	0.259
**ICU stay (days; X** ± **SD)**	7.6 ± 7.9	10.0 ± 3.4	0.113	2.1 ± 4.8	0.3 ± 1.8	**0.014**	7.9 ± 8.3	2.0 ± 2.8	**0.046**
**Pleuritic pain**	19 (13.5)	6 (5.8)	**0.049**	2 (6.5)	5 (6.7)	1.00	17 (15.5)	1 (3.4)	0.121
**Bronchospasm**	66 (46.8)	23 (22.1)	**0.001**	8 (25.8)	17 (22.7)	0.803	58 (52.7)	6 (20.7)	**0.003**
**Infiltrates (X-ray / CT)**	102 (72.3)	47 (45.2)	**0.001**	18 (58.1)	31 (41.3)	0.137	84 (76.4)	16 (55.2)	**0.024**
**Cavitations (X-ray / CT)**	31 (22.0)	7 (6.7)	**0.001**	1 (3.2)	4 (5.3)	0.642	30 (27.3)	3 (10.3)	0.084
**Nodules (X-ray / CT)**	48 (34.0)	22 (21.2)	**0.027**	5 (16.1)	16 (21.3)	0.541	43 (39.1)	6 (20.7)	0.081
**Treatment with Quinolones**	41 (29.1)	42 (40.4)	0.065	12 (38.7)	27 (36.0)	0.827	29 (26.4)	15 (51.7)	**0.009**

### Variables associated with mortality

Table 6 includes variables showing statistical significance in the bivariate analysis comparing dead patients vs. those that survived considering the total population, colonised patients and patients with Aspergillosis. The multivariate analysis performed including the variables shown in the Table arose the following results:

a) Total population The multivariate analysis was statistically significant (p < 0.001, R^2^ Cox = 0.261) and mortality was associated with malignancies (p < 0.001; OR = 4.65, 95% CI = 2.05-10.58), intake of steroids (whether prior or during admission) (p = 0.005; OR = 4.33, 95% CI = 1.55-12.09), antifungal treatment (p < 0.001; OR = 3.68, 95% CI = 1.81-7.49), and the APACHE II score (p < 0.001; OR = 1.14, 95% CI = 1.07-1.20) and negatively associated with COPD (p = 0.045; OR = 0.44, 95% CI = 0.20-0.98).

b) Patients categorised as colonised In the multivariate analysis the logistic regression was significant (p = 0.001; R^2^ Cox = 0.246).Mortality was associated with presence of malignancies (p = 0.001; OR = 31.87, 95% CI = 3.78-258.59) and worsening of X-ray signs (p = 0.002; OR = 25.38, 95% CI = 3.13-205.89), and it was negatively associated with COPD (p = 0.030; OR = 0.11, 95% CI = 0.02-0.81).

c) Patients categorised as with Aspergillosis The multivariate analysis was statistically significant (p < 0.001, R^2^ Cox = 0.290) and mortality was positively associated with worsening of X-ray signs (p = 0.011; OR = 3.04, 95% CI = 1.28-7.21), number of antibiotics post-culture (p = 0.013; OR = 1.51, 95% CI = 1.09-2.08), APACHE II score (p = 0.011; OR = 1.09, 95% CI = 1.02-1.17), and negatively associated with diagnosis of aspergillar tracheobronchitis (p = 0.009; OR = 0.27, 95% CI = 0.10-0.73) and isolation of species other than *A. fumigatus* (p = 0.042; OR = 0.14, 95% CI = 0.02-0.93).

**Table 6:** Significant clinical variables in the bivariate analysis of patients with respect to mortality, [n (%)] except where indicated

	**Total patients (n = 245)**			**Colonised (n = 106)**			**Aspergillosis (n = 139)**		
	**Died (n = 76)**	**Survived (n = 169)**	**p**	**Died (n = 13)**	**Survived (n = 93)**	**p**	**Died (n = 63)**	**Survived (n = 76)**	**p**
**Days to isolation (X** ± **SD)**	13.0 ± 14.2	9.2 ± 8.5	**0.012**	6.5 ± 8.5	7.7 ± 6.3	0.126	14.3 ± 14.8	10.8 ± 10.2	0.153
**Smokers**	17 (22.4)	21 (12.4)	**0.047**	2 (15.4)	12 (12.9)	0.805	15 (23.8)	9 (11.8)	0.063
**COPD**	49 (64.5)	124 (73.4)	0.158	8 (61.5)	65 (69.9)	0.549	41 (65.1)	59 (77.6)	0.101
**Malignancies**	21 (27.6)	17 (10.1)	**0.001**	4 (30.8)	6 (6.5)	**0.015**	17 (27.0)	11 (14.5)	0.089
**Charlson (X** ± **SD)**	3.20 ± 2.64	2.34 ± 1.85	**0.012**	4.1 ± 3.2	2.2 ± 1.7	**0.019**	3.0 ± 2.5	2.5 ± 2.0	0.376
**McCabe (Non fatal)**	33 (43.4)	114 (67.5)	**0.001**	2 (15.4)	63 (67.7)	**0.001**	31 (49.2)	51 (67.1)	**0.032**
**APACHE II (X** ± **SD)**	16.3 ± 7.7	11.7 ± 5.9	**0.001**	14.0 ± 7.9	11.3 ± 5.8	0.191	16.8 ± 7.6	12.3 ± 6.1	**0.001**
**ICU admission**	32 (42.1)	26 (15.4)	**0.001**	4 (30.8)	11 (11.8)	0.086	28 (44.4)	15 (19.7)	**0.002**
**ICU stay (days; X** ± **SD)**	6.9 ± 7.6	6.8 ± 7.6	0.937	1.9 ± 5.8	0.7 ± 2.5	0.167	2.9 ± 6.0	1.5 ± 4.9	**0.017**
**Dyspnoea**	59 (77.6)	114 (67.5)	0.106	6 (46.2)	60 (64.5)	0.201	53 (84.1)	54 (71.1)	0.068
**Infiltrates (X-ray / CT)**	59 (77.6)	90 (53.3)	**0.001**	7 (53.8)	42 (45.3)	0.570	52 (82.5)	48 (63.2)	**0.011**
**Cavitations (X-ray / CT)**	19 (25.0)	19 (11.2)	**0.006**	1 (7.7)	4 (4.3)	0.487	18 (28.6)	15 (19.7)	0.237
**Worsening X-ray signs**	40 (52.6)	18 (10.7)	**0.001**	4 (30.8)	3 (3.3)	**0.002**	36 (57.1)	15 (19.7)	**0.001**
**Steroids prior to admission**	43 (56.6)	58 (34.3)	**0.001**	4 (30.8)	25 (26.9)	0.748	39 (61.9)	33 (43.4)	**0.030**
**Steroids during admission**	68 (89.5)	117 (69.2)	**0.001**	11 (84.6)	63 (67.7)	0.336	57 (90.5)	54 (71.1)	**0.004**
**Antifungal treatment**	60 (78.9)	81 (47.9)	**0.001**	7 (53.8)	24 (25.8)	0.052	53 (84.1)	57 (75.0)	0.187

## Discussion

Three questions arise when physicians face cultures positive to *Aspergillus* from lower respiratory samples in non-immunocompromised/non-neutropenic patients: Is there colonisation or infection?, should the patient be treated with antifungals?, and which is the prognosis?, that is, how to interpret and manage patients from which *Aspergillus* is obtained. The retrospective analysis of this series of 245 immunocompetent patients showing at least two respiratory cultures positive to *Aspergillus*, that were further categorised as colonised, probable or proven aspergillosis, tried to explore how these three questions are addressed in the clinical practice. The diagnostic categorization of patients was based on criteria adapted from those defined by Bulpa et al. for COPD patients because we expected that most of the non-neutropenic, non-transplant patients in our series were this type of patients.

The answer to the first question is important since an early diagnosis is crucial to improve prognosis [1]. It has been postulated that isolation of an *Aspergillus* species from respiratory samples in critically ill patients (even when immunocompetent) should not be routinely discarded as colonisation [3], but in elderly patients (commonly having underlying diseases) isolation is usually interpreted as colonisation [10]. Confirmation of infection obliges the demonstration of histopathological evidence that is not usually feasible in this type of patients [18]. Early administration of antifungals may be life-saving, but overinterpretation of the potential clinical significance of *Aspergillus* isolation may drive to needless treatments, with their associated problems and costs [5,18]. How does clinicians solve the dilemma?

In this study 245 records from patients with at least two respiratory cultures positive to *Aspergillus* were identified, 139 of them (56.7%) classified as with Aspergillosis (probable + proven) following adapted Bulpa et al. criteria. This contrasts with other published series where the percentage of patients showing Aspergillosis was markedly lower [3,5,10], probably due to the at least two positive cultures required as inclusion criteria in our study. In this sense, a recent study in our country reported 22.1% of probable invasive pulmonary aspergillosis among hospitalized COPD patients with at least one positive culture to *Aspergillus*[19]. In addition to signs/symptoms suggestive of aspergillosis as cavitations in X-ray/CT scan, worsening of radiological findings and interaction of COPD and need for O_2_, according to the multivariate analysis performed, stay in the ICU, congestive heart failure and previous intake of steroids were also positively associated with Aspergillosis; all of them factors that had been previously described [1,6,7]. Although clinical signs/symptoms and radiological findings are usually non specific in critically ill patients [3], in our series requests of CT scan and the galactomannan test were significantly more frequent in patients further classified as with Aspergillosis than in those colonised, this suggesting a right clinical suspicion of colonisation/infection by treating physicians.

Initiation of antifungal treatment and time for initiation is a matter of considerable debate. Clinical manifestations of pulmonary aspergillosis may be initially indistinguishable from bacterial bronchopneumonia (fever, cough, purulent sputum) [20]. The recovery of the same *Aspergillus* species from several respiratory samples in the course of antibiotic-resistant pneumonia in patients with risk factors is clearly evocative of the diagnosis [21]. Therefore it has been proposed that the isolation of an *Aspergillus* species from the respiratory tract in critically ill patients with risk factors (COPD after corticosteroids exposure, severe underlying disease) and clinical features of pneumonia indicates prompt consideration of antifungal treatment [3,7].

In our series (with 72.6% of pulmonary aspergillosis among patients with Aspergillosis) antifungal treatment was not initiated in 23.2% patients with probable aspergillosis while it was administered to 29.2% of patients classified as colonised. Administration of antifungals was negatively associated with the presence of severe liver disease, probably because of the renal and hepatic toxicity of antifungals [11]. ICU admission and days in the ICU were associated with prescription of antifungals, both in colonised and aspergillosis patients. Interestingly, in patients with Aspergillosis antifungal treatment was negatively associated with Gold III + IV and treatment with quinolones. The negative association with treatment with wide-spectrum quinolones could probably be linked to the commented similarity of initial clinical manifestations of pulmonary aspergillosis with bacterial bronchopneumonia. In addition this could be the reason for the significantly lower number of severe COPD patients found among treated vs. non-treated patients (65.3% vs. 88.0%) in our series when analysing patients with Aspergillosis. However the percentage of COPD patients with Aspergillosis (Table 5) that received antifungal treatment in our series was 75% (75 out of 100), a percentage similar to the one (71%) described in a published review of 65 cases [22].

Voriconazole was the most frequent antifungal drug, with 74.5% of treated patients with Aspergillosis receiving this drug alone or in combined regimens. For primary treatment of invasive aspergillosis, the triazole voriconazole is recommended for most patients [23] and, although its superiority over lipid formulations of Amphotericin B or echinocandins has not been proved in the subset of COPD patients [21], the superiority of voriconazole versus Amphotericin B has been demonstrated in invasive aspergillosis in immunocompromised patients [24].

The high mortality of invasive pulmonary aspergillosis in non-neutropenic critically ill patients has been attributed, at least partially, to difficulties in timely diagnosis caused by insensitive and non specific clinical signs and lack of unequivocal diagnostic criteria [11] precluding the early needed treatment. In the present study, as expected, significant higher mortality was found in proven than in probable aspergillosis and in the latter than among colonised patients (78.6% vs. 41.6% vs. 12.3%). Interestingly, COPD was negatively associated with mortality in colonised patients but not in patients with Aspergillosis. Higher mortality rates (>90%) in invasive pulmonary aspergillosis than those found in the present study have been reported in previously published series in COPD patients, but including only patients in the ICU [1,22]. Although the strength of the present study resides in the description of clinical features of aspergillosis in a large series of patients with at least two positive lower respiratory samples, several study limitations should be considered. The retrospective nature of the study based on information (clinical, radiological reports…) recorded in clinical records from 29 different hospitals, implies bias derived from inter-hospital differences in patient’s management. This could specially affect the management of patients concomitantly presenting other hospital infections and/or organ dysfunction. In addition most patients in the Aspergillosis category were classified as “probable aspergillosis”, thus making difficult to establish firm conclusions due to the low number of proven cases or patients with CT scan images as halo sign or air crescent sign.

## Conclusions

The analysis of the present series including 245 patients not selected based on clinical diagnoses but with at least two cultures of respiratory samples positive to *Aspergillus* showed that antifungal treatment is not always closely linked to the diagnostic categorisation (colonisation vs. Aspergillosis). Administration of antifungals in patients with Aspergillosis was negatively associated with severe COPD (GOLD III + IV) and concomitant treatment with quinolones, probably due to the similarity of signs/symptoms between this entity and pulmonary bacterial infections.

## Abbreviations

APACHE: Acute physiologic and chronic health evaluation; COPD: Chronic obstructive pulmonary disease; GOLD: Global initiative for chronic obstructive lung disease; ICU: Intensive care unit; NYHA: New York heart association; OR: Odds ratio.

## Competing interests

J.B. and L.A. are members of Sociedad Española de Quimioterapia. J.B. and M-J. G. have received travel grants from Pfizer S.L.U. for presentation of study results in International congresses. Other authors: none to declare.

## Authors' contributions

Conceived and designed the study: JB, J-JG, M-JG and LA. Collection of data: JB, BA, EM, FG-L, JD, DdC, VV, MH-F and F-JG-P. Analyzed the data: J-JG, M-JG and LA. Wrote the paper: JB, M-JG and LA. Made substantial intellectual contributions and approved the manuscript: all authors.

## Additional members of the ASP Investigator Group who contributed data

I. Cuesta (Complejo Hospitalario de Jaén, Jaén), R. Sanchez-Silos (Complejo Hospitalario Universitario de Badajoz, Badajoz), R. Pascual and E. Lopez (H. Universitari de Bellvitge, l'Hospitalet de Llobregat), F. Perez-Grimaldi (H. de Jerez, Jerez de la Frontera), I. Campos (H. Universitario de Gran Canaria Dr. Negrín, Las Palmas de Gran Canaria), A. Pallares, A. Fernandez-Villar and I. Iglesias (H. Xeral de Vigo, Vigo), J. Blanquer, M.L. Briones and D. Navarro (H. Clínico Universitario de Valencia, Valencia), G. Fernandez-Calleja, J.R. Hernández, L. Martinez-Martinez (H. Universitario Marqués de Valdecilla, Santander), P. Llinares, M. Vares, D. Velasco (Complejo Hospitalario de A Coruña, A Coruña), M.A. Sepulveda and E. Heredero (H. Virgen de la Salud, Toledo), S. Merlos (H. Universitario Virgen de las Nieves, Granada), A. Flor, I. Serra, R. Blavia and D. Estivill (H. Althaia Xarxa Assistencial, Manresa), E. Sanchez-Haya (H. de Donosti, San Sebastián), V. Acha and X. Beristain (H. Virgen del Camino, Pamplona), M. Chanza, T. Lloret and F. Sanz (H. General Universitario de Valencia, Valencia), C. del Valle and J.R. Maestre (H. Central de la Defensa Gomez Ulla, Madrid), M. Segarra (H. General de Elda, Elda), P. Serra (H. Universitari Germans Trias i Pujol, Badalona), R. Lama and M. Cause (H. General Universitario Reina Sofia, Cordoba), M. Riera, L. Martin-Pena, C. Marinescu and N. Borrell (H. Universitari Son Dureta, Palma de Mallorca), R. Malo and M. Valle (H. Universitario Puerta de Hierro, Majadahonda), E. Nuño and A. Infante (H. Universitario Virgen de la Victoria, Malaga), P. Hernandez (H. Universitario Nuestra Señora de Candelaria, Sta. Cruz de Tenerife), A. Ruedas, F. Hidalgo (H. Universitario Ramón y Cajal, Madrid), L. Vigil and A. Garcia-Garcia (Instituto Nacional de Silicosis, Oviedo), A. Robles (H. Universitario La Paz, Madrid) and J. Prieto (Univ. Complutense, Madrid).

## Pre-publication history

The pre-publication history for this paper can be accessed here:

http://www.biomedcentral.com/1471-2334/12/295/prepub
